# Detection of VIM-1, VIM-2 and IMP-1 metallo- β-lactamase genes in *Klebsiella pneumoniae* isolated from clinical samples in Sanandaj, Kurdistan, west of Iran

**Published:** 2019-06

**Authors:** Nasrin Bahmani

**Affiliations:** Zoonoses Research Center, Research Institute for Health Development, Kurdistan University of Medical Sciences, Sanandaj, Iran

**Keywords:** *Klebsiella pneumoniae*, Carbapenem-resistant, *bla*_VIM_, *bla*_IMP_

## Abstract

**Background and Objectives:**

*Klebsiella pneumoniae* is an important cause of serious nosocomial infections among Gram-negative bacteria. The aim of this study was evaluating the prevalence of VIM-1, VIM-2, and IMP-1 metallo-β-lactamase genes in clinical specimens at two teaching hospitals in Sanandaj, Kurdistan west of Iran.

**Materials and Methods:**

Four hundred different clinical specimens were collected from hospitalized patients or referred to hospitals from May 2013 to March 2014 in Sanandaj, Kurdistan, Iran. MBLs – producing *K. pneumoniae* detected by Double Disk Synergy Test. The MBL positive isolates were examined for the presence of VIM-1, VIM-2 and IMP-1 genes using PCR technique.

**Results:**

Of four hundred clinical specimens, 114 *K. pneumoniae* isolates were identified. Twenty-eight (24.6%) isolates were resistant to imipenem and 15 strains (53.6%) were positive for MBL enzymes production. PCR results showed VIM-1 and IMP-1 genes frequencies are 4 (26.7%) and 1 (6.7%). Only one strain of *K. pneumoniae* was found to be MBL producer among the outpatients.

**Conclusion:**

The study results exhibited a high level of resistance to most of the antibiotics tested and high prevalence of MBLs producing in *K. pneumoniae* at two hospitals. Thus, the infection control methods and the implementation of antibiotic agents should be taken into account.

## INTRODUCTION

*Klebsiella pneumoniae* is a Gram-negative bacillus belonging to the Enterobacteriaceae family, which is the major cause of nosocomial and opportunistic infections namely pneumonia, urinary tract infections, bacteremia, and wound infections across the world, with significant morbidity and mortality rates (1). High prevalence of antibiotic resistance in *Enterobacteriaceae*, especially in *K. pneumoniae* has been reported in Iran which is challenging for hospitals. The carbapenems such as imipenem, meropenem are used to treat infections caused by Gram-negative bacteria when there is resistance to other antibacterial agents. The presence of carbapenem-resistance in Gram-negative bacteria in both the community constitutes and hospital environments are a prominent development in the field of infectious disease management and control (2).

One of the most important ways to become resistant to imipenem is production of metallo-beta-lactamases (MBL). These enzymes belong to group 3 of Bush classification based on their substrate and inhibitor profiles and to Ambler class B beta-lactamases according to their amino acid sequence homology (3, 4). Several types of MBL have been identified in Gram-negative bacteria, which are geographically widespread including Verona integron-encoded metallo-β-lactamase (VIM), New Delhi metallo-β-lactamase (NDM) and imipenemase (IMP). The first reports on MBLs were VIM-1 in Italy and IMP-1 in Japan (5). During the last few years incidence rates of MBL production of the members of Gram-negative organisms especially, *Acinetobacter baumannii*, *Pseudomonas aeruginosa* and *Enterobacteriaceae* (mainly *E. coli* and *K. pneumoniae*) has been significantly increased and are the cause of life-threatening infections among human (6). Recently, there are several reports on the emergence of VIM-producing *K. pneumoniae* (VPKP) isolates across the world. VIM types (e.g. VIMI,VIM2) MBLs in Southern Europe and the Far East, Australia, North and South America, India and Iran, and IMP-type MBLs in Southeast Asia are predominant (7). There are several phenotypic methods for detection of MBL-producing bacteria. The ability of inhibitors (EDTA, Thiol compounds, and Dipicolinic acid) to control the activity of these enzymes are the foundation of these methods such as double-disk synergy test (DDST) using EDTA with IPM or ceftazidime (CAZ) (8), the Hodge test (9) and the MBL E-test (10). For identification of Gram-negative bacteria MBLs, PCR assay was described in 1996 (11).

Thus, the knowledge about the antimicrobial susceptibility patterns and MBLs-producing *K. pneumoniae* is helpful for the treatment of infections caused by these strains. The purpose of this study was to determine the prevalence of VIM-1, VIM-2 and IMP-1 genes of MBLs-producing in *K. pneumoniae* isolates in hospitalized patients or referred to Hospitals in Sanandaj, Kurdistan west of Iran.

## MATERIALS AND METHODS

### Bacterial isolates and identification.

In this study, four hundred clinical isolates which were collected from different clinical specimens in hospitalized patients or referred (outpatients) to Beasat and Toohid hospitals during May 2013 to March 2014 in Sanandaj, Kurdistan, Iran. The included samples were as urine (n=208), wound (n=52), blood (n=50), tracheal aspiration (n=48) and sputum (n=42). The isolated bacteria were identified according to standard methods including colonial morphology, Gram staining, catalase, oxidase, urease and IMVIC tests (12). Detection and final approval of testing bacterial isolates was based on the use of API20E kit (Bio-Merieux, Marcy-l’Etoile, France).

### Antibacterial susceptibility testing.

Antibacterial susceptibility testing was performed using the disk diffusion method according to the guidelines of the Clinical and Laboratory Standards Institute (CLSI) documents (13). The antibiotics used comprised of piperacillin (100 μg), ceftazidime (30 μg), cefotaxime (30 μg), cefazoline (30 μg), tetracycline (30 μg), kanamycin (30 μg), Imipenem (10 μg), and Meropenem (10 μg) (Himedia, Mombay, India). Minimum inhibitory concentration (MIC) for imipenem was determined by E-test method (AB Biodisk, Solna, Sweden) for all *K. pneumoniae* isolates according to CLSI guideline (14).

### Detection of MBLs.

The presence of MBL production was screened by the double-disk synergy test (DDST) using imipenem (IPM) disk and imipenem in combination with EDTA (6). After overnight incubation, the difference in ≥7mm between the inhibition zone diameter of the IPM-EDTA disk and IPM the only disk was considered to be a positive for the presence of MBLs interpreted as being DDS test. In this study, *K. pneumoniae* ATCC 700603 was used as positive control.

### Molecular identification of *bla*_VIM1_, *bla*_VIM2_, *bla*_IMP1_ genes.

DNA was extracted using boiling method (15). Briefly, 0.5 ml of distilled water with 1 loop of bacteria was removed from an overnight culture (Trypticase soy agar, Merck, Germany) and the cell suspension was heated for 15 min at 100°C, and then centrifuged at 13,000 g for 5 min. The supernatant obtained was used as template for amplification. Polymerase chain reaction (PCR) for the detection of *bla*_IMP-1_, *bla*_VIM-1_, and *bla*_VIM-2_ genes was carried out on a thermal cycler (Bio-Rad, USA) (16). PCR products were electrophoresed on 1.5% agarose gel and photographed by UV trans-illuminator. Primers and conditions of polymerase chain reaction are shown in Table 1.

**Table 1.** Primers and conditions of polymerase chain reaction in *K. pneumoniae*

**Primer Name**	**Primer Sequence (5′ to 3′)**	**Cycle**	**Initial Denaturati**	**Cycling**	**Final Extension**	**Length (bp)**
IMP-1	F: ACCGCAGCAGAGTCTTTGCC R: ACAACCAGTTTTGCCTTACC	33	94°C 5 min	94°C 30 s, 56°C 30 s, 72°C 1 min	72°C, 7 min	587 bp
VIM	F: CAAGTCCGTTAGCCCATTCC R: GGCACAACCACCGTATAGCAC	33	94°C 5 min	94°C 30 s, 57°C 30 s, 72°C 1 min	72°C, 10 min	539 bp
VIM2	F: ATTGGTCTATTTGACCGCGTC R: TGCTACTCAACGACTGAGCG	33	94°C 5 min	94°C 30 s, 56°C 30 s,72 °C 1 min	72°C, 10 min	780 bp

## RESULTS

**Fig. 1.**
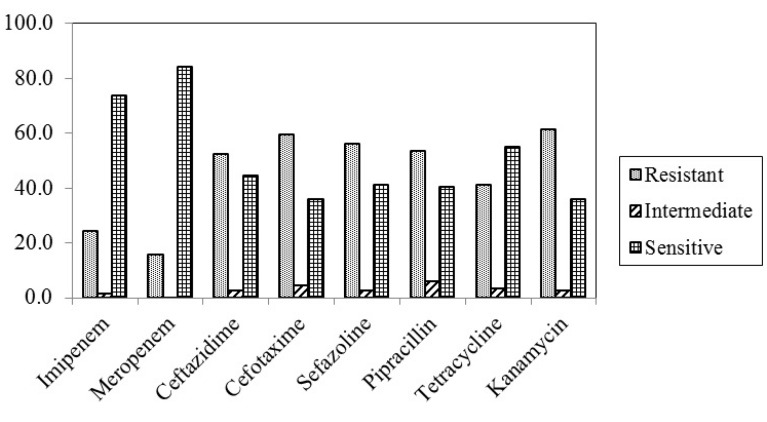
Pattern of antibiotic use in *K. pneumonia*

This cross-sectional study was performed on four hundred clinical samples collected from hospitalized patients and outpatients in Toohid and Beasat hospitals in Sanandaj, Kurdistan, Iran. From four hundred clinical samples, 190 *E. coli*, 114 *K. pneumoniae*, 64 *P. aeroginosa*, 20 *Enterobacter* spp, 10 *Proteus* spp, and 2 *Klebsiella oxytoca* were identified. From 114 *K. pneumoniae* isolates (28.5%), 77 isolates (67.5%) belong to patients who were hospitalized and 37 (32.5%) isolates belonged to outpatients. The distribution of *K. pneumoniae* in hospitalized patients pertaining to the type of wards shown in Table 2. The frequency of *K. pneumoniae* isolates in outpatients included; urine 25 (67.6%), wound 9 (24.3%) and sputum 3 (8.1%). The most and the least number of *K. pneumoniae* isolates belonged to ICU (28.9%) and CCU (5.3%), respectively. The study population included 54% male and 46% female patients with an age range between 2 and 80 years. Among 114 *K. pneumoniae* isolates, 28 (24.6%) and 18 (15.9%) were resistant to imipenem and meropenem, respectively. Resistant to kanamycin was detected among 61.4% of isolate in disk diffusion method (Fig. 1). *K. pneumoniae* resistant to antibacterial agents in hospitalized patients and outpatients are shown in Table 3.

**Table 2.** Frequency of *K. pneumoniae* isolates in different samples and units in hospitalized patients

**Sample**	**Urine**	**Tracheal**	**Wound**	**Sputum**	**Blood**
**Unite**					
ICU	12 (40%)	10 (55.6%)	5 (41.7%)	3 (33.3%)	3 (37.5%)
Surgery	7 (23.3%)	4 (22.2%)	6 (50%)	0	1 (12.5%)
Internal	6 (20%)	4 (22.2%)	1 (8.3%)	6 (66.7%)	3 (37.5%)
CCU	5 (16.7%)	0	0	0	1 (12.5%)
Total	30 (39%)	18 (23.3%)	12 (15.6%)	9 (11.7%)	8 (10.4%)

**Table 3.** Antibiotic resistance of *K. pneumoniae* isolated from hospitalized patients and outpatients

**Antibacterial agents**	**N (NO%)**	**Hospitalized patients N (NO%)**	**Outpatients N (NO%)**
Imipenem	28 (24.6)	22 (28.5)	6 (7.8)
Meropenem	18 (15.9)	15 (19.5)	3 (8.1)
Cephotaxime	68 (59.6)	49 (63.8)	19 (51.4)
Ceftazidime	60 (52.6)	46 (59.7)	14 (37.8)
Cefazoline	64 (56.1)	37 (48)	27 (35)
Pipraciline	61 (53.5)	40 (52)	21 (27.2)
Tetracycline	47 (41.2)	32 (41.6)	15 (40.5)
Kanamycin	70 (61.4)	60 (77.9)	10 (27)

**Fig. 2.**
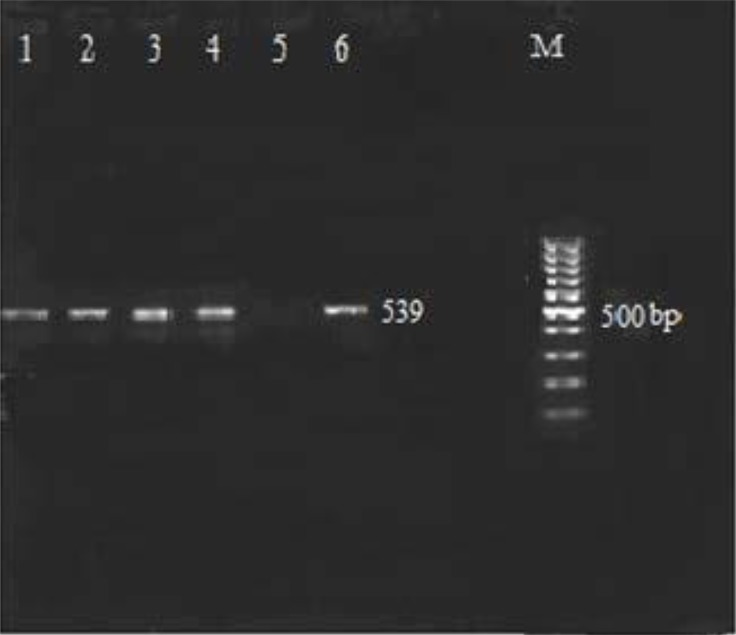
PCR analysis for the *bla*_VIM1_ gene in *K. pneumoniae* lane1, 2, 3, 4 have VIM1 gene; Lane 5 negative control (distilled water), lane 6 positive control *K. pneumoniae* ATCC 700603 and Lane M: DNA Ladder 100 bp

**Fig. 3.**
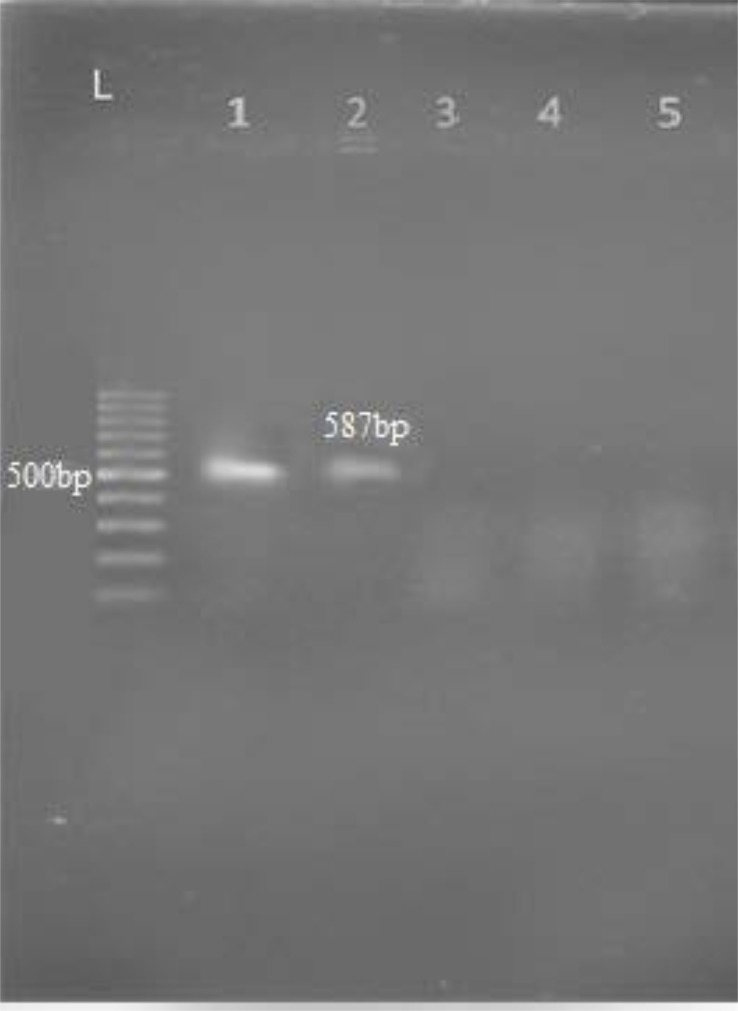
PCR analysis for the *bla*_IMP_ -1gene in *K. pneumoniae* Lane 1: positive control *K. pneumoniae* ATCC 700603, Lane 2: IMP-1gene Lane 3: Negative control (distilled water). Lane L: DNA Ladder 100 bp

Upon examining the frequency of MBL enzymes pertaining to *K. pneumoniae* isolates, 28 of 114 isolates (24.6%) were resistant to imipenem and 15 cases (53.6%) were positive by Double Disk Synergy Test (imipenem disk alone and imipenem with-EDTA). MIC (E-test) by IPM strips was performed for all isolates. All VIM-1 and IMP-1 producing isolates of *K. pneumoniae* had MIC > 4 μg/ml. The VIM-1 produces were resistant to 4 antibiotics, cefotaxime, cefazoline, ceftazidime, and kanamycin. PCR results showed 4 isolates (26.7%) were positive for *bla*_VIM-1_ (Fig. 2) and 1 isolate (6.7%) were positive for *bla*_IMP-1_ (Fig. 3), none of tested isolates were positive for *bla*_VIM-2_ gene.

Higher prevalence of VIM-1 producing isolates of *K. pneumoniae* was seen in urine samples (20%). Only one VIM-1 producing *K. pneumoniae* isolates found in outpatient urine was detected by PCR. Distribution of metallo-β -lactamases in clinical samples are shown in Table 4.

**Table 4.** Distribution of *K. pneumoniae* MBLs production (VIM1, IMP1) in clinical samples

	**Urine**	**Tracheal**	**Sputum**	**Blood**	**Wound**	**Total**
Metallo-β-Lactamase	7 (46.6%)	4 (26.7%)	1 (6.7%)	1 (6.7%)	2 (13.3%)	15
VIM-1	3 (20%)	1 (6.7%)	0	0	0	4
IMP-1	1 (6.7%)	0	0	0	0	1

## DISCUSSION

*K. pneumoniae* is a member of the *Enterobacteriaceae* family. The quantity of reports shows that the isolation of species of this genus from both hospital and community samples has increased (17). In the current study, from total clinical samples, prevalence of *K. pneumoniae* was 28.5%, which is close to the study in Brazil (26.2%) (18), but it was higher in comparison with the study in Iran (25.3%) (19), and a study in India (25%) (20). In this study, *K. pneumoniae* showed varying levels ranging from susceptibility and resistance towards the tested antibiotics. Antibiotic resistance profiles of the isolates using disk diffusion method indicated that resistance to imipenem was 24.6% that was similar to Ghaffarian study (23.3%) in 2018 (21) as well as Khodadadian (25.6%) in 2018 (19), but it was lower than the finding of other studies conducted by Rajabnia (52%) in 2015 (22) Lorenzoni (29.7%) in 2018 (23). Our results proved that meropenem and imipenem remain effective drugs against *K. pneumoniae* isolates. In the present study, more than half of *K. pneumoniae* isolates were resistant to other important antimicrobial agents such as kanamycin (61.4%), cefotaxime (59.6%), cefazoline (56.1%) and ceftazidime (52.6%) which matched the results achieved in the north of Iran in 2016 (21).

Extensive variation in the prevalence and antibiotic resistance is probably due to the variation in risk factors, the widespread use of antibiotics for the treatment of infections and differences in geographical locations. Some of the important reasons for increasing drug resistance in Iran are the lack of communication between physicians and microbiologists, limited infection control programs and poor sanitation. The results of this study proved comparatively low resistance to the majority of *K. pneumoniae* isolates in the outpatients when compared to the patterns from hospitalized patients. These findings may imply that these drugs still be useful for treating uncomplicated cases where the organisms are indicated as etiologic agents of diseases in the hospital. In the present study, the highest number of *K. pneumoniae* were isolated from urine samples in hospitalized patients which admitted to ICU, and also maximum resistance to imipenem was observed in urine samples, which were accordance with studies of Rai in 2011 (17). ICU has been known as a source of creating, spreading and enhancing antimicrobial resistance and patients are exposed to a number of potential risk factors for colonization/infection such as high invasive procedures, elderly people, longterm hospitalization and misuse of suitable antibacterial agents, and *Klebsiella* spp. is one of the most common pathogens isolated in ICUs (24).

In the past few years, MBLs have been identified via clinical isolating by increasing frequency. The enzymes produced by these strains (*K. pneumoniae*) have been responsible for prolonged nosocomial out-breaks and a serious threat to public health world-wide (25).

In regards to MBL enzymes in this study (53.5%) was in line with a study in India in 2012 (26), but it was higher compared to a study in Malaysia in 2015 (27). The results of this study showed that the majority of MBL producing *K. pneumoniae* (14/15) were extracted from hospitalized patients and only one isolate (1/15) was MBL producer in outpatients by DDST. The presence of MBL-producing bacteria in the outpatients portends serious health risk because the organisms that produce MBL are notably resistant to carbapenems including imipenem and meropenem, which are antibiotics reserved for serious bacterial infections (28). In this study, VIM-1 gene prevalence was 26.6% (4/15) which matched with the results achieved in India in 2013 (29), but it was higher than the study published in Iran in 2018 (19), and in India in 2014 (20), and it was lower compared to studies in Nepal 2014 (6). By DDST and PCR analysis, we determined one IMP-1 producing *K. pneumoniae* (6.7%) in urine samples in hospitalized patients, which was similar to findings of Khodadadian study in 2018 (19), but it was lower than study reported by Jin Young in 2016 (30). It seems that the difference in the prevalence of MBL-producing *K. pneumoniae* strains in different hospitals is due to the genetic difference between species, spreads to resistance genes and affects the spread patterns of different MBLs in various countries.

In our study, the MICs (E-test) of imipenem against 4 VIM-1 and IMP-1 Metallo-β lactamase producing *K. pneumonaie* isolates ranged from 4 to 32 mg/l and exhibited IPM resistant phenotypes. The resistance to imipenem may also be attributed to the loss of porins. In the present study, MBL positive isolates (5 strains) were resistant to at least 4 antibiotics including cefotaxime, cephazolin, ceftazidime, and kanamycin. In a study by Rastegar Lari et al. among 19 *K. pneumonaie* isolates resistant to imipenem, 9 were resistant to all other antibiotics (2). Our data emphasizes the need to implement appropriate strategies to control the infection to detect MBL-producing strains in numerous hospitals, which can be an effective step to reduce the incidence of such as genes. In our study *bla*_VIM-1_ and *bla*_IMP-1_ were identified in *K. pneumonaie* isolates and studies have shown that other mechanisms of resistance may interfere in the MBL phenotypic detection. Thus, other carbapenemase producing genes should be investigated. The results of this study showed although the carbapenems had the best activity among the tested antibiotics, there is a high level of resistance in *K. pneumoniae* to most of the other tested antibiotics and the majority MBL production of *K. pneumoniae* was associated with hospitalized patients in ICU. Therefore, accurate and rapid detection of these MBL-producing strains is vital among isolates circulating in high-risk wards such as ICUs, for suitable selection of effective therapy and nosocomial infection control.
